# AlN Production in Co-Flow Filtration Mode at Low Pressures

**DOI:** 10.3390/ma14195482

**Published:** 2021-09-22

**Authors:** Nikolay Evseev, Pavel Nikitin, Mansur Ziatdinov, Ilya Zhukov, Alexei Vakutin

**Affiliations:** 1 Institute for Problems of Chemical and Energetic Technologies of the Siberian Branch of the Russian Academy of Sciences, 659322 Biysk, Russia; gofra930@gmail.com (I.Z.); alex-wakutin@mail.ru (A.V.); 2 National Research Tomsk State University, 36 Lenin Ave., 634050 Tomsk, Russia; upavelru@yandex.ru (P.N.); ziatdinovm@mail.ru (M.Z.)

**Keywords:** aluminum nitride, filtration combustion, co-flow filtration, self-propagating high-temperature synthesis, phase composition

## Abstract

In this work, the process for obtaining aluminum nitride in the combustion mode of co-flow filtration of a nitrogen–argon mixture was investigated. The combustion of granules consisting of aluminum and aluminum nitride as an inert diluent was studied under conditions of co-current filtration in a flow of nitrogen and a nitrogen–argon mixture in the range of a specific flow rate of 1.5–5.0 cm^3^/(s∙cm^2^). It was found that the specific flow rate of the gas mixture and the amount of argon in the nitrogen–argon mixture had a significant effect on the rate and the temperature of combustion. The structure and phase composition of the synthesis products were studied. The maximum achieved yield of the AlN phase was 95 wt.%. Moreover, this method is energy efficient and allows the production of metal nitrides without the use of high-pressure reactors.

## 1. Introduction

Aluminum nitride is a unique compound of high applied value in the field of microelectronics, in particular, for creating substrates for hybrid microcircuits based on high thermal conductivity ceramics [1]. Due to its high thermal conductivity and low coefficient of thermal expansion, aluminum nitride is one of the promising materials for the production of such substrates [2]. To obtain AlN ceramics with high thermal conductivity, the initial powder must satisfy a number of conditions, such as chemical purity, shape, and particle size distribution of particles. The thermal conductivity of AlN decreases due to the presence of impurities such as iron, silicon, and oxygen [3,4]. Studies have shown that the oxygen impurity has the greatest effect on the thermal conductivity of AlN ceramics. In this case, to achieve high thermal conductivity, the oxygen impurity should not exceed 1.0 wt.% [5]. Another factor affecting thermal conductivity is the density of ceramics, which depends on the shape and size of the particles [6]. There are many methods for producing aluminum nitride, the main of which are: the carbothermal [7], the plasma-chemical [8], and the gas-phase method [9]. The above technologies for producing AlN are characterized by high energy consumption. In contrast, there is the self-propagating high-temperature synthesis (SHS) method [10,11,12,13,14]. Studies devoted to the synthesis of aluminum nitride by the SHS method have long been conducted [15,16]. The SHS method is a highly efficient method for producing aluminum nitride, but it requires the use of high-pressure reactors.

Traditionally, self-propagating high-temperature synthesis and filtration combustion of metal powders in nitrogen are studied under conditions of natural filtration in high-pressure reactors [17]. Another type of filtration combustion is known: when the reagent gas is forced into the reaction chamber, moving along with the combustion front through the reaction products. A number of theoretical works [18,19,20,21] are devoted to this method; however, despite this, there are few experimental works on the combustion of metals and alloys in the co-flow filtration mode. Basically, such works [22,23] are devoted to the study of the co-flow combustion of a mixture of titanium and carbon powders in the nitrogen and/or argon atmosphere. This method is of high practical importance, since it is energy efficient and allows the production of metal nitrides without the use of high pressures. It should be noted that the composition of the initial products, as well as the operating parameters (the composition and flow rate of the gas mixture), significantly affect the combustion characteristics and, as a consequence, the reaction products. Thus, it is promising to study the features of the combustion of metal powders in a nitrogen-containing gas flow. In this work, we studied the effect of the specific flow rate of the gas mixture on the rate and combustion temperature of aluminum, the structure, and the phase composition of the materials obtained during the synthesis of aluminum nitride in the co-flow filtration mode.

## 2. Materials and Methods

### 2.1. Raw Components

The raw components were aluminum powder with an average size of 8 μm and a purity of 98% and AlN powder (purity of 98%) with an average size of 25 μm, previously obtained by the SHS method. The combustion of the powders was carried out in a nitrogen/nitrogen–argon mixture. The purity of argon and nitrogen was 99.99%.

### 2.2. Experimental Part

Combustion in the co-flow filtration mode was carried out in a SHS reactor, which consisted of a reaction chamber (inner diameter of 1.6 cm), a gas supply unit, and a unit for recording operating parameters. The experimental procedure on the combustion of the raw components in the co-flow filtration mode is similar to the experimental procedure on the combustion of chromium powder in the co-current gas flow, which was described in detail in our work [24].

Preliminary experimental work has shown that the initial powders are strongly compacted when gas is supplied, which negatively affects the filtration process. Thus, to prevent such an effect, the raw components were granulated. The granulation process was carried out as follows. The raw powders were mixed with 1% polyvinyl alcohol. The obtained mixture was rubbed through sieves with a mesh size of 0.5 mm and 1 mm, respectively. The obtained granules were dried in a vacuum furnace at 150 °C and repeatedly rubbed through the sieves to obtain granules with a size of 500–1000 µm.

In this work, the combustion process of a sample consisting of aluminum and aluminum nitride granules was investigated in the mode of co-current filtration in a flow of nitrogen and the nitrogen–argon mixture in the range of a specific flow rate of 1.5–5.0 cm^3^/(s∙cm^2^). It is known that the completeness of combustion is inversely proportional to the exothermicity of the composition [19]. For the SHS method, the main way to increase the completeness of combustion is to dilute the metal with the reaction product [19]. According to the preliminary experimental work, it was found that the combustion temperature of aluminum in the co-flow filtration combustion mode significantly exceeds the melting temperature of aluminum. In this case, the melting process significantly affects the filtration process, and, consequently, the obtained experimental results. Due to the high exothermicity of aluminum, to increase the completeness of combustion, as well as to prevent melting, aluminum nitride was added to the aluminum powder as an inert diluent. To determine the optimal composition of the granules, experimental studies were carried out. The following ratios were taken as the initial compositions of the granules: 100 wt.% Al; 30 wt.% AlN + 70 wt.% Al; 50 wt.% AlN + 50 wt.% Al; 70 wt.% AlN + 30 wt.% Al (hereinafter the “wt.” index will be omitted). It should be noted that the combustion of 100% Al; 30% AlN + 70% Al; 50% AlN + 50% Al led to the presence of melts in the combustion products, which impeded the filtration process and led to combustion breakdown. In the general case, the experiment was carried out as follows.

**Figure 1:**
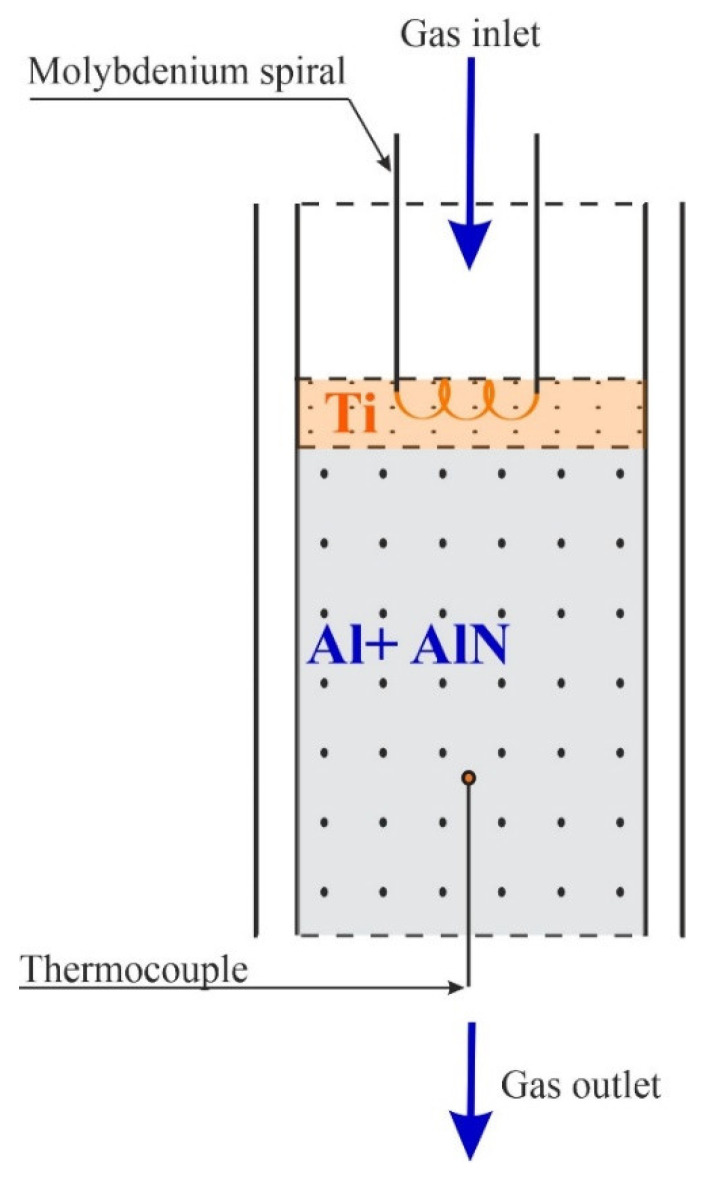
Scheme of the flow-through SHS reactor.

Granules with a layer thickness of 4 cm were poured into the reaction chamber (Figure 1). To ignite the sample, titanium granules with a size of 500–1000 µm were used. In this case, titanium powder also acted as an oxygen-obtainer. The thickness of the ignition layer was 4 mm (Figure 1). The initiation of the reaction was performed by applying an electric pulse to the spiral, which was brought into contact with the surface of the igniting composition. After ignition, a combustion front was formed, which propagated along the sample. As a result of the combustion process, a sample was obtained, which was removed from the reaction chamber, and ground by hand in a mortar into a powder for further research.

### 2.3. Characterization

The pressure was monitored using a manometer and pressure sensors at the inlet and the outlet of the reactor. The gas flow rate at the inlet and the outlet of the reactor was controlled using electronic micro-flow meters of the hot-wire type “Red-y”. The reaction temperature was measured with a WR5/20 tungsten-rhenium thermocouple. Data from sensors and thermocouples were displayed on-line and recorded in a computer.

The particle size distribution of the raw powders was determined using a FRITSCH Analysette 22 MicroTec plus (Germany) analyzer by laser diffraction. X-ray phase analysis was performed using a Shimadzu 6000 diffractometer with CuKα radiation based on the PDF-4 database. The microstructure of the raw powders and combustion products was determined using a QUANTA 3D microscope equipped with an energy-dispersive attachment (EDX).

## 3. Results and Discussion

### 3.1. Determination of the Optimal Composition of the Initial Powder Mixture

**Figure 2:**
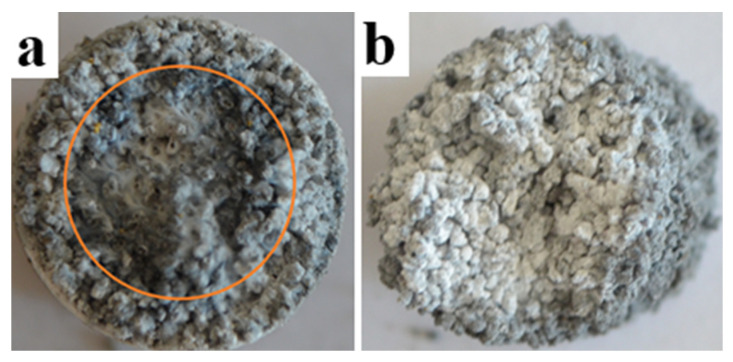
Appearance of the combustion products: (**a**) 50% AlN + 50% Al; (**b**) 70% AlN + 30% Al.

Figure 2 shows images of combustion products of AlN-Al mixtures in various concentrations obtained in the co-flow filtration mode in a nitrogen atmosphere with a specific flow rate of 3.0 cm^3^/(s∙cm^2^).

As can be seen from Figure 2a, the central region of the (50% AlN + 50% Al) sample was partially melted, which makes the filtration process difficult. In contrast, in the (70% AlN + 30% Al) sample shown in Figure 2b, this region was not observed. The melting of the central part was explained by the highest combustion temperature in the center of the sample in comparison with the temperature at the periphery, where there was heat exchanged with the wall of the reaction chamber.

According to the obtained results, it was found that the optimal ratio of Al to AlN, at which it is possible to avoid the effect of melting on the co-flow filtration process, was 70% AlN + 30% Al.

### 3.2. Influence of the Specific Nitrogen Flow Rate on the Combustion Rate of the Initial Composition

One of the main operating parameters that has a significant effect on the combustion process in the co-flow filtration mode is the specific flow rate of the gas mixture.

**Figure 3:**
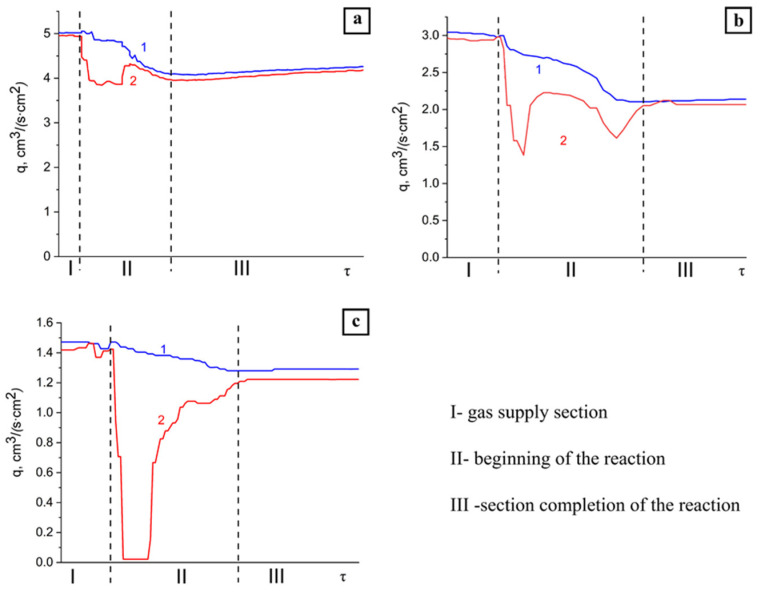
Profile patterns of the specific N2 flow rate obtained during combustion of the (70% AlN + 30% Al) sample. Parameters: (**a**) q = 5.0 cm^3^/(s∙cm^2^), (**b**) q = 3.0 cm^3^/(s∙cm^2^), (**c**) q = 1.5 cm^3^/(s∙cm^2^), 1–reactor inlet, 2–reactor outlet.

The combustion of the granular mixture of aluminum and aluminum nitride in the range of specific nitrogen flow rate of 1.5–5.0 cm^3^/(s∙cm^2^) was studied. Figure 3 shows the profile patterns of the specific nitrogen flow rate at the inlet and the outlet of the reactor.

As can be seen from Figure 3, profiles of the specific flow rate have a complex shape, which can be conditionally divided into three parts: I-a gentle section (gas supply section), II-a section with a sharp drop in the specific flow rate (the beginning of the reaction), and III-a section with a monotonic increase in the specific flow rate (the completion of the reaction). The specific flow rate in region III increased monotonically; however, its value did not reach the initial value. It is possible to achieve a stable propagation of the combustion wave at the specific flow rate of more than 1.5 cm^3^/(s·cm^2^). Figure 3 shows that during combustion at q = 5.0 and 3.0 cm^3^/(s∙cm^2^), the specific flow rate at the outlet decreased sharply but did not reach zero; thus, the gas was in excess. In this case, one part of the gas was absorbed, and the other part passed through the reaction products. At the specific flow rate of 1.5 cm^3^/(s∙cm^2^), an almost complete absorption of the reaction gas was observed. A further decrease in the specific flow rate led to a lack of fuel and the termination of the AlN synthesis reaction.

At the specific flow rate of 3.0 and 5.0 cm^3^/(s∙cm^2^), two cavities were observed in region II, the presence of which was obviously associated with different rates of absorption of the reaction gas by the igniting composition and the initial composition. It should be noted that after the completion of the reaction, the specific flow rate did not reach its initial value. This can be explained by the following: during the reaction, the granular layer was compacted as a result of thermal sintering, and the passage of gas through the powder layer in the initial amount became impossible.

**Figure 4:**
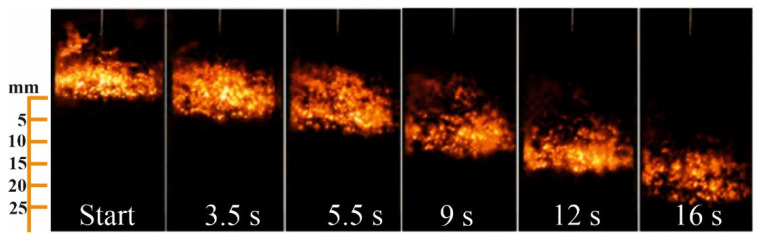
Combustion front propagation in the co-flow filtration mode with the parameters: 70% AlN + 30% Al, q = 3.0 cm^3^/(s∙cm^2^), N_2_ = 100%.

Figure 4 shows typical photographic records of the combustion front propagation in the co-flow filtration mode in a nitrogen atmosphere at the following parameters: 70% AlN + 30% Al, q = 3.0 cm^3^/(s∙cm^2^). The combustion rate was measured experimentally over the thickness of the sample. It was found that the combustion front propagates at a constant speed through the entire thickness of the sample. Therefore, the combustion rate was determined as the ratio of the sample thickness to the burning time.

Table 1 shows the combustion rate of the initial composition depending on the value of the specific nitrogen consumption. As can be seen from Table 1, the higher the specific flow rate of nitrogen, the higher the maximum combustion rate.

### 3.3. Influence of the Specific Flow Rate of Nitrogen on the Combustion Temperature of the Initial Composition

**Figure 5:**
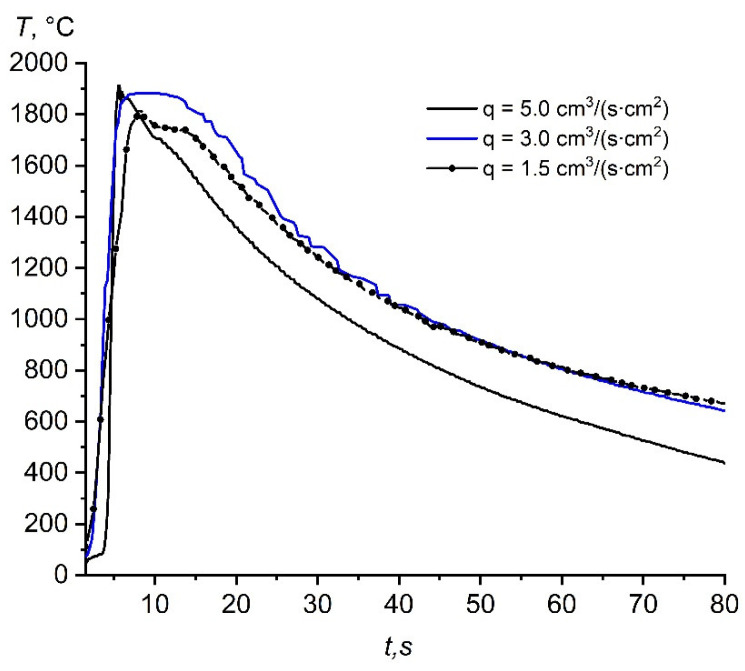
Heat patterns obtained during the combustion of the (70% AlN+ 30% Al) sample in nitrogen.

Figure 5 shows the heat patterns obtained during the combustion of the (70% AlN + 30% Al) sample depending on the specific flow rate of nitrogen.

The maximum combustion temperature of the initial composition depending on the specific flow rate of nitrogen is shown in Table 2.

As can be seen from Table 2, the higher the specific flow rate of nitrogen, the higher the temperature and the combustion rate. Obviously, this was due to the larger amount of fuel entering the reaction zone. At the same time, a more rapid cooling of the reaction products was observed: the “quenching” process [24,25]. Such quenching fixes the composition of the products formed in the high-temperature region [25]. It should be noted that an increase in the combustion temperature with an increase in the specific flow rate of nitrogen was also observed, but at these operating parameters it was insignificant, since the exothermicity of the initial composition was significantly reduced by the addition of 70% AlN.

### 3.4. Influence of the Argon Addition to the Reaction Gas on the Combustion Characteristics of the Initial Composition

The second most important operating parameter that had a significant effect on the combustion process in the co-flow filtration mode was the composition of the gas mixture. It is known that the co-flow filtration mode is very sensitive to the purity of the filtering reagent [19]. Previous experiments [24,25] confirmed that the addition of inert argon to nitrogen significantly affects the combustion process. The more cold ballast gas (argon) enters the reaction zone, the more energy will be spent on heating it and the more intensively the heat will be redistributed. Thus, the combustion of the initial composition in the co-flow of a nitrogen–argon mixture with a 15% argon content was studied.

#### 3.4.1. Influence of the Specific Flow Rate of the Nitrogen–Argon Mixture on the Combustion Rate of the Initial Composition

**Figure 6:**
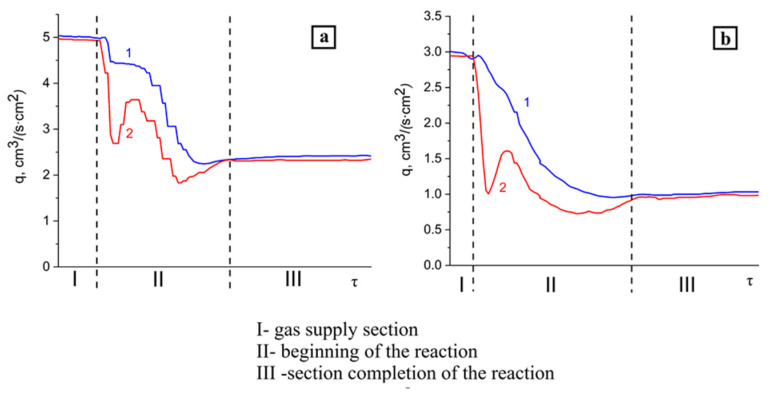
Profile patterns of the specific flow rate of the nitrogen–argon mixture obtained during combustion of the (70% AlN + 30% Al) sample. Parameters: Ar = 15%, (**a**) q = 5.0 cm^3^/(s∙cm^2^), (**b**) q = 3.0 cm^3^/(s∙cm^2^), 1–reactor inlet, 2–reactor outlet.

With the addition of 15% argon to nitrogen, it was not possible to carry out combustion at the specific flow rate of 1.5 cm^3^/(s∙cm^2^) since this led to the breakdown of combustion. Therefore, the combustion of the initial composition in the nitrogen–argon atmosphere was studied at the specific flow rate of 3.0–5.0 cm^3^/(s∙cm^2^). Figure 6 shows the profile patterns of the specific flow rate of the nitrogen–argon mixture at the inlet and the outlet of the reactor obtained during the combustion of the (70% AlN + 30% Al) sample.

Both in the case of combustion of the initial composition in the nitrogen flow (Figure 3) and in the case of combustion in the flow of the nitrogen–argon mixture (Figure 6), the profiles of the reaction gas flow rate can also be conditionally divided into three areas. An increase in the specific N_2_-Ar flow rate from 3 to 5 cm^3^/(s∙cm^2^) led to an increase in the combustion rate (Table 3).

The combustion rate in the nitrogen–argon mixture at the specific flow rate of 3.0 cm^3^/(s∙cm^2^) decreased by more than two times. However, with an increase in the specific flow rate to 5.0 cm^3^/(s∙cm^2^), the difference between the combustion rates in nitrogen and the nitrogen–argon mixture was only 0.23 mm/s.

#### 3.4.2. Influence of the Specific Flow Rate of the Nitrogen–Argon Mixture on the Combustion Temperature of the Initial Composition

**Figure 7:**
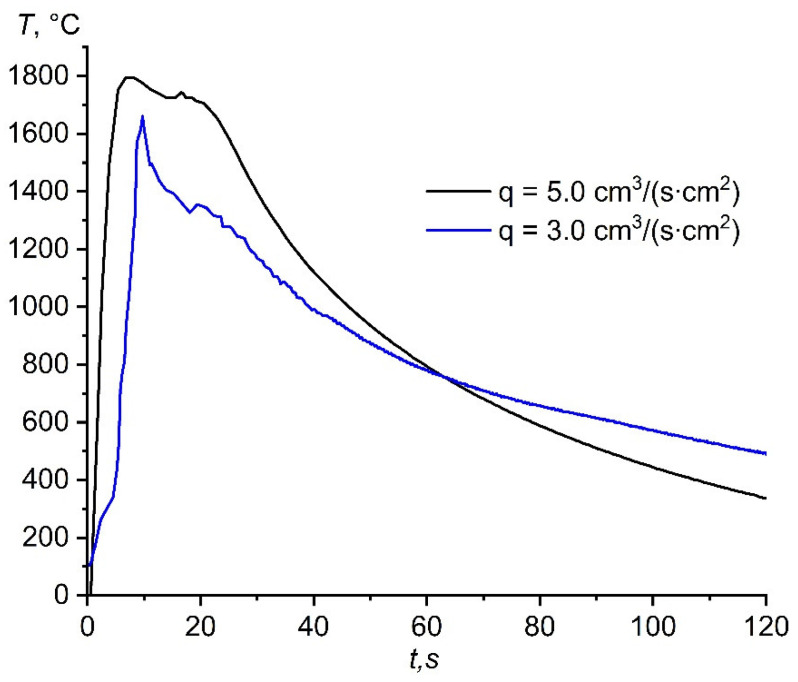
Heat patterns obtained during the combustion of the (70% AlN+ 30% Al) samples in the flow of the nitrogen–argon mixture.

Figure 7 shows a comparison of the heat patterns obtained during the combustion of the (70% AlN + 30% Al) samples in the flow of the nitrogen–argon mixture, depending on the specific flow rate.

An increase in the specific flow rate of the nitrogen–argon mixture from 3 to 5 cm^3^/(s∙cm^2^) led to an increase in the maximum temperature (Table 4).

**Figure 8:**
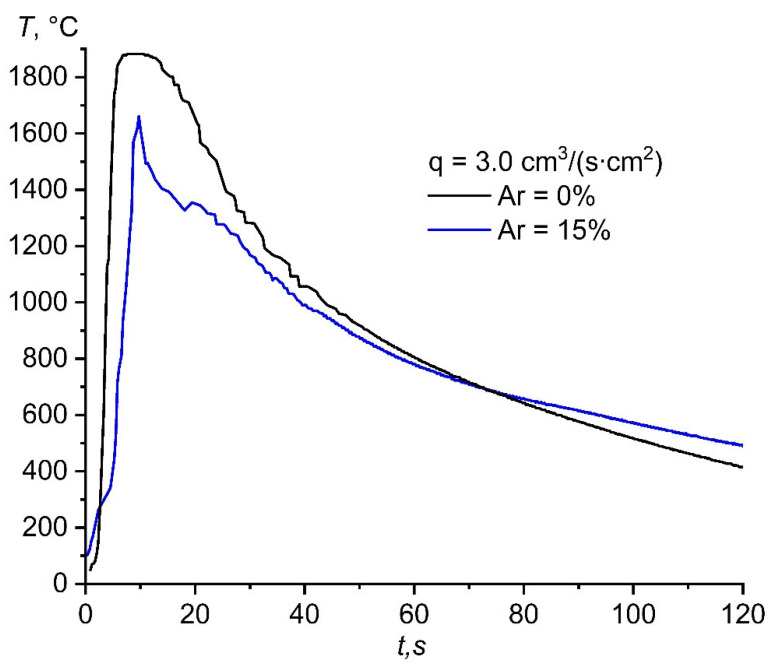
Heat patterns obtained during combustion of the (70% AlN + 30% Al) sample in the flow of nitrogen and nitrogen–argon mixture.

Figure 8 shows a comparison of the heat patterns during the combustion of the initial composition in the nitrogen flow and in the nitrogen–argon mixture. In the case of combustion in the flow of the nitrogen–argon mixture (Figure 7), an increase in the combustion temperature was observed; however, the combustion temperature was significantly lower than the combustion temperature in pure nitrogen at the same specific flow rate (Figure 8). A decrease in the maximum combustion temperature in the nitrogen–argon mixture was associated with the redistribution of heat for heating cold argon. With an increase in the specific flow rate of the nitrogen–argon mixture, an increase in the temperature and combustion rate was observed. Obviously, this effect is still associated with a larger supply of fuel to the reaction region and the simultaneous slip of argon through the reaction products, in which already-heated argon transfers heat to the region behind the combustion front, heating it up. However, this effect is observed due to the high exothermicity of the composition. Apparently, when the critical value of the argon concentration in the mixture is reached, a decrease in the combustion temperature will be observed with an increase in the specific flow rate, since heating a large content of cold argon will require a significant amount of heat.

### 3.5. Phase Composition of Combustion Products

#### 3.5.1. Phase Composition of the Combustion Products Obtained in the Nitrogen Flow

To calculate the weight fractions of phase components in the combustion products, the Rietveld method was used.

**Figure 9:**
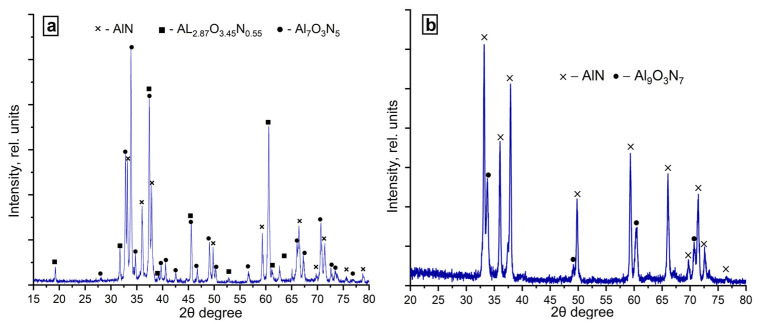
XRD patterns of the combustion products obtained from the (70% AlN+ 30% Al) sample in the nitrogen flow: (**a**) q = 1.5 cm^3^/(s∙cm^2^) and (**b**) q = 5.0 cm^3^/(s∙cm^2^).

XRD patterns of the combustion products obtained from the (70% AlN + 30% Al) sample in the nitrogen flow are shown in Figure 9.

Phase analysis of the combustion products obtained in the nitrogen flow showed that at the specific flow rate of 1.5 cm^3^/(s∙cm^2^), in addition to the main phase of aluminum nitride, there were phases of aluminum oxynitride (Table 5). Moreover, there was an oxynitride phase with a high nitrogen content (according to energy dispersive analysis, 22–25 wt.%). Presumably, this was due to less sharp cooling of the combustion products. At the flow rate of 5.0 cm^3^/(s∙cm^2^), the products contained 95% aluminum nitride and only 5% aluminum oxynitride Al_9_O_3_N_7_. The content of the AlN phase was higher at the nitrogen flow of 5.0 cm^3^/(s∙cm^2^), which was explained by an excess of fuel.

#### 3.5.2. Phase Composition of the Combustion Products Obtained in the Nitrogen–Argon Flow

**Figure 10:**
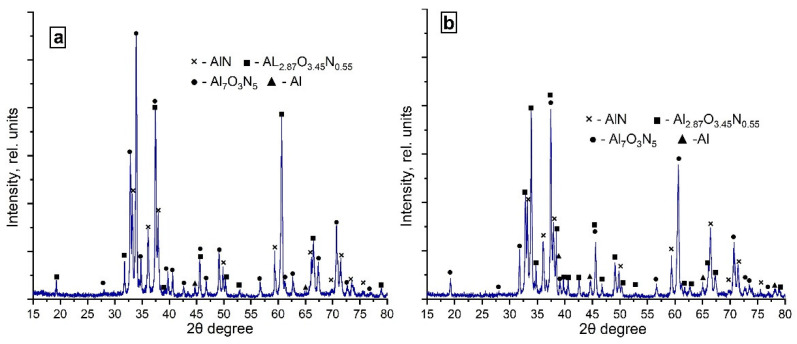
XRD patterns of the combustion products obtained from the (70% AlN+ 30% Al) sample in the nitrogen–argon flow: (**a**) Ar = 15%, q = 3.0 cm^3^/(s∙cm^2^) and (**b**) Ar = 15%, q = 5.0 cm^3^/(s∙cm^2^).

XRD patterns of the combustion products obtained from the (70% AlN + 30% Al) sample in the nitrogen–argon flow are shown in Figure 10.

After combustion in the nitrogen–argon mixture, the main phases in the combustion products were aluminum nitride and aluminum oxynitride (Table 6). Moreover, at the specific flow rate of 3.0 cm^3^/(s∙cm^2^), the Al_2.87_O_3.45_N_0.55_ phase prevailed among the detected oxynitride phases, and, at the specific flow rate of 5.0 cm^3^/(s∙cm^2^), the Al_7_O_3_N_5_ phase prevailed. Apparently, this was also due to the smaller amount of fuel that entered the reaction area. Free aluminum was found in an insignificant amount (2%) at the flow rate of 5.0 cm^3^/(s∙cm^2^) and less than 1% at the flow rate of 3.0 cm^3^/(s∙cm^2^), which was probably due to a more rapid cooling of the combustion products as a result of which the aluminum particles did not react.

As can be seen from the obtained results, the complete conversion of aluminum nitride in the studied range of the specific flow rate and the small diameter of the reaction chamber (1.6 cm) was not achieved. Obviously, this was due to two factors. First, there was a temperature gradient at which the maximum temperature was concentrated in the center of the sample, and the minimum temperature was at the periphery, as a result of heat loss due to the contact of granules and the walls of the reactor. Thus, presumably, to reduce the effect of the thermal gradient, it was necessary to increase the diameter of the reaction chamber. Second, this is the “quenching” mode, in which the incoming gas cools the combustion products and “fixes” the reaction products, while during combustion in the natural filtration mode there is a nitrogen-addition phase [25]. Thus, the degree of purity of the AlN phase during synthesis in the co-flow filtration mode is determined by the purity of the initial powders and the combustion parameters (the specific flow rate and the composition of the reaction mixture).

### 3.6. The Structure of Combustion Products

The synthesized samples are easily destructible products of a white-gray color. Grinding of the combustion products led to obtaining granules of the initial size, and further grinding of the granules led to obtaining particles comparable to the size of the initial powder.

**Figure 11:**
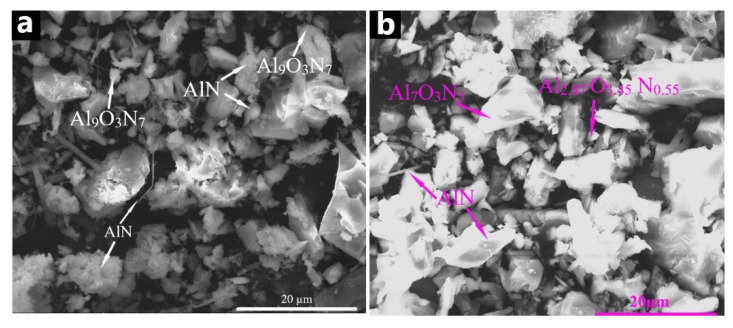
The structure of the combustion products obtained from the (70% AlN + 30% Al) samples with the parameters: q = 3 cm^3^/(s∙cm^2^), (**a**) nitrogen flow, (**b**) nitrogen–argon flow.

Figure 11 shows the structure of the combustion products obtained in the flow of nitrogen (Figure 11a) and the nitrogen–argon mixture (Figure 11b) at the same gas flow rate. As can be seen from Figure 11, there were particles of aluminum nitride with a whisker-shape, acicular (rod-like), layered structure, as well as sharply angular particles. However, the structure of aluminum nitride after combustion in the nitrogen flow was mainly represented by acicular particles, which are particles with a layered structure and whiskers up to 20 µm in length; the diameter of whiskers did not exceed 0.3 µm. The structure of aluminum nitride after combustion in the nitrogen–argon mixture was represented mainly by sharply angular particles and acicular particles. Whiskers of aluminum nitride were contained in small amounts, and their average length reached 5 µm; the diameter did not exceed 0.1 µm. Apparently, the higher content of particles with a whisker structure was due to the higher synthesis temperature in the nitrogen flow.

## 4. Conclusions

In this work, the combustion of the granular mixture consisting of (70% AlN + 30% Al) was studied. The combustion products were studied, and the fundamental possibility of obtaining aluminum nitride of various compositions in the co-flow filtration mode by varying the operating parameters was shown. For the first time, the effect of the specific flow rate of the gas mixture on the rate and temperature of aluminum combustion in the co-current gas flow was experimentally studied. It was revealed that the specific consumption of the gas mixture, as well as the composition of the nitrogen–argon mixture, has a significant effect not only on the combustion rate and the temperature but also on the structure of the combustion wave, which manifests itself in the form of temperature profiles, which ultimately affect the combustion products. The maximum achieved purity of the AlN phase was 95 wt.%. The structure of aluminum nitride is represented mainly by acicular (rod-like) particles, particles with a layered structure and whiskers up to 20 µm in length; the diameter of the whiskers did not exceed 0.3 µm. The method makes it possible to obtain metal nitrides without using high-pressure reactors. During the synthesis, the maximum pressure at the outlet of the reactor did not exceed 1.3 atm, which is an order of magnitude lower than the pressures used in the production of aluminum nitride by the traditional SHS method. The choice of the optimal combustion parameters, the geometric parameters of the reaction chamber (diameter), and the use of high-purity raw powders will increase the purity of the combustion products.

## Figures and Tables

**Table 1:** Combustion rate of the sample (70% AlN + 30% Al) depending on the specific flow rate of nitrogen.

q, cm^3^/(s∙cm^2^)	υ, mm/s
1.5	0.7
3 5	1.45 1.5

**Table 2:** Maximum combustion temperature of the (70% AlN + 30% Al) sample depending on the specific flow rate of nitrogen.

q, cm^3^/(s∙cm^2^)	T_max_, °C
1.5	1831
3 5	1860 1915

**Table 3:** Combustion rate of the (70% AlN+ 30% Al) sample.

Ar, wt.15%	
q, cm^3^/(s∙cm^2^)	υ, mm/s
3 5	0.68 1.27

**Table 4:** Maximum combustion temperature of the (70% AlN + 30% Al) samples.

Ar, wt.15%	
q, cm^3^/(s∙cm^2^)	T_max_, °C
3 5	1661 1792

**Table 5:** Weight fraction of phases in the combustion products (the nitrogen flow).

q, cm^3^/(s∙cm^2^)	Phase	Weight Fraction, %
1.5	AlN Al_2.87_ O_3.45_ N_0.55_ Al_7_O_3_N_5_	77 15 8
5.0	AlN Al_9_ O_3_ N_7_	95 5

**Table 6:** Weight fraction of phases in the combustion products (the nitrogen–argon flow).

q, cm^3^/(s∙cm^2^)	Phase	Weight Fraction, %
3.0	AlN Al_2.87_ O_3.45_ N_0.55_ Al_7_O_3_N_5_ Al	72 17 11 <1
5.0	AlN Al_2.87_ O_3.45_ N_0.55_ Al_7_O_3_N_5_ Al	74 7 17 2
